# Intestinal Host-Microbe Interactions under Physiological and Pathological Conditions

**DOI:** 10.4061/2010/386956

**Published:** 2010-09-29

**Authors:** Rodrigo Bibiloni, Eduardo J. Schiffrin

**Affiliations:** ^1^Ruakura Research Centre, AgResearch Ltd., East Street, Private Bag 3123, Hamilton 3240, New Zealand; ^2^HealthCare Nutrition, Research and Development, Nestlé Nutrition, Nestec Ltd., Avenue Reller 14, 1800 Vevey, Switzerland

## Abstract

The intestinal mucosa is unique in that it can be tolerant to the resident, symbiotic microbiota but remaining, at the same time, responsive to and able to fight pathogens. The close interaction between host-symbiotic microbiota at the mucosal level poses important challenges since microbial breaches through the gut barrier can result in the breakdown of gut homeostasis. In this paper, hosts-integrated components that help to preserve intestinal homeostasis including barrier and immune function are discussed. In addition global alterations of the microbiota that can play a role in the initiation of an exaggerated inflammatory response through an abnormal signaling of the innate and adaptive immune response are briefly described.

## 1. Introduction

The close association of dense bacterial communities of the gut with the mucosa at different habitats of the distal intestine are the basis of symbiotic interactions between the host and its microbiota. Host factors encourage the establishment of the microbiota preserving at the same time tissular homeostasis. The magnitude and the diversity of intestinal microflora represent yet a threat of microbial break through the single-cell epithelial layer that covers the intestinal surface [1]. The invasion of host tissue by resident bacteria would certainly result in the breakdown of the symbiotic host-microbiota interactions.

Commensal invasion of the intestinal tissues is a rare event during the homeostatic situation or occurs in a very limited and controlled manner [2–4]. In some major disturbances of gut balance and integrity it results in severe clinical conditions such as bacteraemia, necrotising enterocolitis in the newborn period, or chronic local or systemic inflammatory conditions.

A diversity of components contributes to the preservation of the barrier integrity. Cellular and extracellular host components at the intestinal mucosa participate in the prevention of bacterial leakage from the lumen. Working in concert with the barrier function, the immunological tolerance to commensals sustains the symbiotic microbiota-host interactions.

The intestinal immune system is tolerant to the components of the commensal microbiota, however immunological tolerance is not the result of immunological ignorance. In fact commensals stimulate host reactions that do not promote host tissue damage but rather cytoprotective mechanisms at the mucosal environment [5, 6]. Thus, systemic immune tolerance concurs with local host responses to commensal bacteria that strengthen the barrier function maintaining the microbiota within the intestinal environment through mechanisms that do not stimulate inflammation. This is the result of permanent interactions between the host and its commensal microbiota [7].

A basic functional feature of the intestine immune system is to avoid tissue-damaging overreactions to commensals that would unnecessarily damage intestinal tissues by way of inflammatory processes, at the same time the intestinal barrier and the innate immune defense needs to be effective regarding the distinct recognition of commensals from pathogens.

Microbial-associated molecular patterns (MAMPs) or “infectious nonself” [8–11] are broadly shared molecular motifs expressed on most bacteria either commensal or pathogens. They interact with pattern recognition receptors (PRRs), expressed on epithelial cells or innate mucosal immune cells such as macrophages and dendritic cells [12]. PRRs are germ-line encoded molecules that are expressed on the plasma membrane or in intracellular endosomal compartments, that is, Toll-like receptors (TLRs) [11], or cytosolic molecules such as nucleotide-binding oligomerization domain (NOD) [13].

The best characterized PRRs are the family of TLRs but, as mentioned above, other families of PRRs have been described. Together they can sense the presence of bacteria in the extracellular and intracellular compartments [11, 13, 14].

Several MAMPs that are ligands for PRRs are common to pathogens and commensal microorganisms and yet, the intestine initiates a protective response to the former while allowing an important and complex microbiota to establish itself at the intestinal surface without any detrimental effect on homeostasis. Discrimination between pathogens and commensals clearly involves other mechanisms. The expression and engagement of PRRs in different cellular or different plasma membrane compartments [15], as well as the presence of additional “danger signals” from stressed or damaged tissues, may be important determining factors [16]. Certainly, most pathogens express a number of virulence determinants such as adherence to and invasion of host cells, and the production of toxins. An important virulence trait of pathogens like *Salmonella* and *Shigella *is their capacity to access the intracellular environment where they interact with cytosolic PRRs that are part of the inflammasome molecular complex [14, 16].

In this paper we will discuss the mechanisms that prevent immune/inflammatory reactions against commensals as well as evidences of deviations to this that contribute to IBD. We will focus on (1) mechanisms that limit direct bacterial contact with epithelial cell surfaces either by a secreted mucus layer or the fast clearance of bacterial cells, (2) bacterial dysbiosis commonly associated to pathological conditions, and (3) the modulation of the innate/inflammatory reaction to commensals.

## 2. Intestinal Mucosal Factors Involved in Host-Microbiota Homeostasis

Large quantities of bacteria reside in the gut lumen without initiating any detrimental inflammatory response [17] whereas low numbers of bacteria in blood or tissues trigger an energetic inflammatory response.

**Figure 1:**
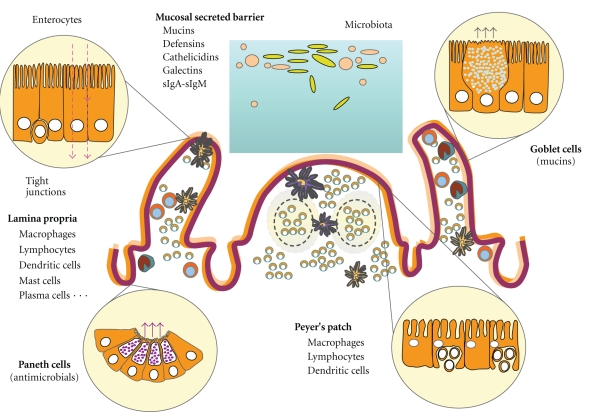
Components of the gut barrier.

The intestinal homeostasis is achieved through robust cellular and molecular mechanisms that contribute to reinforce the intestinal barrier function through secreted products, promote cytoprotective epithelial responses, and innate immune reactions to commensals for clearance of physiological passage of bacteria and modulation of detrimental inflammatory response (Figure 1). 

### 2.1. Mucosal Secreted Barrier

On the luminal side of the epithelial cells, a series of interacting factors work together for the prevention of mucosal bacteria close juxtaposition to host tissues.

One of the first physical contacts between the host and the luminal bacteria is the intestinal mucus layer, which covers the mucosal surface. This mucus is a product of goblet cells that actively secrete the mucin glycoproteins. The secreted barrier is composed primarily by highly glycosylated multimeric glycoproteins produced by goblet cells which are responsible for the viscosity of the mucus [18], but also by other secreted compounds like phospholipids, lectins, immunoglobulins, and antimicrobial peptides (defensins, lysozyme, and cathelicidins).

#### 2.1.1. Mucins

Increasing evidences from animal models show that intestinal inflammation could result from defects in this physical interface (both the secreted and the cellular barriers), even in the presence of normal microbiota and normal innate and adaptive immunity [19–21]. Therefore, it is not surprising that an important area of research on inflammatory bowel diseases has focused on failures in the intestinal barrier function in order to elucidate whether these dysfunctions are primary contributors to the inflammation or a consequence of the inflammatory reaction [22]. This, however, has not been an easy task. The investigation of the secreted barrier is troubled with technical sampling problems; most studies fail to preserve the architecture of the mucus layer [23]. Similarly, the definition of the mucosa-associated bacteria and the location of the microbes in the mucus layer remain controversial because the procedure to collect the biopsies lacks consistency from one study to another, as it has been previously indicated [24]. For instance, the preparation of the subject for the colonoscopy can impact the composition of the bowel community [25], the preparative fluid may still be present in the bowel making difficult to distinguish what is actually being collected [26], bacteria from the outer mucus layer can be easily dislodged and lost during the preparation of the sample [25], and so forth. It seems that at least the inner mucus layer remains quasisterile [27, 28] with most of the microbes residing at least 800 *μ*m from the surface of the mucosal epithelial cells [29].

It has been suggested that the contribution of the intestinal bacteria to the pathogenesis of inflammatory bowel disease could be by increased penetration in the mucus, increased adherence to epithelial cells, or invasion of the epithelium. Schultsz et al. [30] studied the spatial distribution of bacteria in the mucosa of rectal specimens from IBD patients and controls. They observed that bacteria were localised within the mucus layer but did not adhere to epithelial cells and were not present in the lamina propria. They conclude that the intestinal mucus in IBD patients is less protective against endogenous bacteria than in healthy individuals, which could explain the increased association of luminal bacteria with the mucus layer [31]. The reduced protection of the mucus could be a result of a genetically determined alteration, for example, in the glycosylation of glycoproteins that renders the mucus prone to degradation, or a depletion of specific mucin subspecies [32, 33]. Various changes of the mucus properties have been documented since mid 80s [28, 34–37]. Jacobs and Huber observed that ulcerative colitis patients had an altered glycosylated mucin [38]. Fyderek and colleagues showed that the thickness of the mucus layer of adolescents with IBD was three times thinner in both CD and UC patients compared to controls [39]. In ulcerative colitis, there is a decreased mucin sulfation [34].

#### 2.1.2. Antimicrobial Peptides

The antimicrobial peptides provide protection from intestinal infections and contribute to the maintenance of enteric homeostasis; the concentrations of the different products show a decreasing gradient within the mucin gel from the epithelial side to the luminal side [22].

Paneth cells and other mucosal cell types such as enterocytes, colonocytes, and goblet cells are the source of antimicrobial peptides [40]. The importance of these compounds in the susceptibility to mucosal infections has been demonstrated using experimental models [41]. There are two major groups of antimicrobial peptides in humans and other mammals: defensins and cathelicidins [42] which are also active in cell signaling.

The *α*-, *β*-, and *θ*-defensins kill bacteria by membrane disruption [42]. The cysteine-rich *α*-defensins also known as cryptdins [43] are produced as an inactive precursor that requires activation by matrilysin in the small intestine [44]. Experimental models show that *α*-defensins contribute to host defence by influencing the composition and limiting the numbers of resident microbes [45] and in rodents have microbicidal activity against *Escherichia coli*, *Staphylococcus aureus,* and *Salmonella typhimurium*. The nucleotide-binding oligomerization domain-containing protein 2 (NOD2) controls the expression of a distinct subsets of *α*-defensins and defensin-related cryptdins by Paneth cells [46] upon bacterial ligand recognition. It has been shown that some patients with IBD suffer from an impaired synthesis of *α*-defensins [47] associated with NOD2 variants [13].

Expression of *β*-defensins HBD 2 and 3 is induced in the case of inflammation or infection [48]. The induction is mediated by proinflammatory cytokines like IL-1*β* and bacterial signaling through the activation of TLRs. Thus, extracellular and intracellular cell signaling are both involved in the stimulation of bactericidal product secretion by Paneth and superficial epithelial cells.

Cathelicidins, the other main family of antimicrobial peptides, are characterized by an N-terminal signal peptide (cathelin prosequence) and a structurally variable cationic peptide at the C-terminus [41, 42, 49]. The human mature cathelicidin is called LL-37, and it is originated from a precursor molecule that requires proteolytic activation. The processed peptide has antimicrobial activity against Gram-negative and Gram-positive bacteria [50]. It is expressed by gastric epithelial cells, distal small bowel enterocytes, and throughout the colon.

Another homeostatic product that controls microbiota overall size and composition is the production of regenerating islet 3 gamma (Reg III *γ*), a C-type lectin with bactericidal properties [51]. In fact, Reg III *γ* and the human homolog HIP/PAP bind to peptidoglycan component of bacteria particularly of Gram-positive bacteria resulting in bacterial killing. Reg III *γ* expression depends on epithelial stimulation through TLRs ligands to epithelial [5]. It is possible that Reg III *γ* fills a particular antibacterial niche because it targets Gram-positive bacteria such as *Enterococcus fecalis* [51].

The maintenance of intestinal homeostasis depends also on host-microbial interactions that involve protein-carbohydrate recognition [52]. Some animal lectins function as PRR, in the same way that TLRs and NODs do. They cover a wide range of host-microbial interactions. Soluble- and membrane-associated lectins mediate interactions with microorganisms that may lead to mutualistic interactions (commensalism or symbiosis), host colonization, immune recognition by the host, or “subversion” of the nonself recognition functions [52].

Galectins, a subtype of lectins, appear to play important functions in the innate and adaptive immune response at the intestinal mucosa. They are expressed by dendritic cells, macrophages, mast cells, natural killer cells, gamma/delta T cells, and B1 cells, as well as cells from the adaptive immune system (activated B and T cells) [53]. Although galectins lack a typical secretion signal peptide, they are present not only in the cytosol and the nucleus, but also in the extracellular space. Galectin 1 displays anti-inflammatory activities by blocking or attenuating signalling events that lead to leukocyte recruitment, migration, and infiltration [53]. It has been shown that galectin-1 can drive the differentiation of DCs with a regulatory phenotype and function; in fact they can induce T cell tolerance, blunt TH-17 and TH1 responses and suppress autoimmune inflammation through mechanisms involving IL-27 and IL-10 [54].

Galectins can interact directly with bacterial surface glycans in the lumen of the gut or more specifically in the mucus environment. A recent observation has underlined the capacity of galectins to kill bacteria in the lumen of the gut [55]. Both Gram-positive bacteria, such as *Streptococcus pneumoniae*, and Gram-negative bacteria, such as *Klebsiella pneumoniae*, *Neisseria meningitidis*, *Neisseria gonorrhoeae*, *Haemophilus influenzae*, and *Pseudomonas aeruginosa*, display surface carbohydrate galectin ligands [52].

Many human pathogens express on their surfaces diverse carbohydrate structures, and many of these structures have similarities to human antigens, a mechanism utilized by both commensal and pathogens to render themselves immunologically inert. However galectins give the host the opportunity to overcome the pathogenic strategy. *In vitro* and *in vivo* results demonstrate that Gal-4 and Gal-8 possess the ability to specifically kill bacteria expressing blood group antigens. For example, they recognize and kill *Escherichia coli *expressing human blood group antigens while failing to alter the viability of other *E. coli *strains or other Gram-negative or Gram-positive organisms [56]. The ability of Gal-4 and Gal-8 to also kill *α*-Gal-expressing bacteria shows that galectin-mediated killing is not limited to human blood group antigen-expressing bacteria and suggests that galectins may affect the composition of multiple populations of intestinal bacteria, thereby modulating the intestinal microbiota.

#### 2.1.3. Secretory Antibodies

The third mechanism for the luminal sequestering of symbiotic bacteria involves secretory antibodies in particular secretory immunoglobulin A (sIgA). sIgA-coated bacteria have lower chances to become associated with the intestinal epithelial surface. Suzuki et al. have shown that secretions of IgA are as or more important than innate antimicrobial peptides in the regulation of commensal bacterial flora. In fact in induced cytidine deaminase (AID) deficiency in mice, the absence of hypermutated IgA results in two different consequences: (a) a production of large amounts of unmutated IgM and (b) higher colonization of segmented filamentous bacteria of the intestine. In turn this abnormal level of colonization results in a strong and abnormal stimulation of the mucosal immune system [57].

Hypermutated IgA specific for components of the microbiota involves bacterial sampling by DC and a limited migration of DC up to the mesenteric lymph nodes where B cells are induced to differentiate into plasma cells [7, 58]. Lamina propria plasma cells produced dimeric IgA that is shuttled to the apical side of the enterocytes by the polymeric immunoglobulin receptor or secretory component.

### 2.2. The Cellular Barrier

Despite the multiple components of the secreted barrier to prevent bacterial contact with the epithelial layer occasional breaches are inevitable. Thus a second layer of the intestinal immune protection is the rapid detection and clearance of bacteria that penetrate the epithelial layer or go beyond it and into the lamina propria.

Bacterial invasion of epithelial cells or breach of the epithelial barrier provides a signal to epithelial cells to initiate responses that are of inflammatory nature in the majority of the cases and whose final goal is the clearance of invading microorganisms. The epithelial layer seems to play a critical role in the recognition between commensals and pathogens. It is essential that the immune system recognizes and reacts to bacterial associated “danger signals” but remains tolerant to nonthreatening microbes and host cells [12], and the epithelial layer plays an important role.

Nonpathogenic bacteria induce a limited immune reaction on enterocytes, with only a transient innate component [15] that may contribute to the physiological, low-level inflammation in the intestine. In addition they induce the secretion of homeostatic cytokines in the mucosal microenvironment [59]. In contrast, true pathogens induce a rapid and more aggressive response that involves intracellular signaling pathways that detect cellular injury, also called “danger signals” [12]. Taken together, the host response at the epithelial layer can thus be considered as a two-tiered process which in a first instance, involves proinflammatory genes that are triggered by most bacteria, pathogenic or not. Subsequent activation of a second cluster of genes is defined by specific virulence traits of the microorganism [10]. In fact the epithelium has a central role in the initiation of the inflammation in response to bacterial pathogens. For example IL-8 derived from epithelial cells initiates the inflammatory response and tissue damage. However this is necessary for the clearance of invading microorganisms [60] by neutrophils.

A limited number of commensals can physiologically breach the epithelial barrier. When this happens they are taken up and killed by lamina propria macrophages. LP macrophages can clear bacteria without triggering a strong inflammatory reaction [61]. In addition upon bacterial breaching of the epithelial barrier, LP macrophages migrate and produce growth factors for the epithelial restitution [62]. The lamina propria macrophages that express low CD14 are a major cellular component of the mucosal homeostasis.

Another cell type that interacts with commensal bacteria are the dendritic cells [63]. A population of DC—CX3CR1+ DCs—are closely associated with the epithelial lining. In fact CX3CR1+ DCs extend transepithelial dendrites into the gut lumen to sample and process luminal Ags including components of the microbiota [3, 4]. In addition CX3CR1+ DCs initiate the host defence to intestinal pathogens, such as *Salmonella*, as shown by the enhanced susceptibility of CX3CR1-deficient animals to Salmonella infection [64].

The interactions of DCs with T cells mediate the initiation of the adaptive immune response. In addition to lamina propria DC, a rare population of white blood cells have a crucial role in determining the nature of immune reactions and in fine-tuning the balance between immunologic tolerance or induction of inflammation upon recognition of commensals or pathogens, respectively. This functional dualism is crucial at the intestinal level where the immune system does not react against commensals but should be still reactive to clear pathogens upon challenge. A long-standing question has been how dendritic cells drive these distinct immune outcomes required for the clearance of pathogens. Recently, CD103+ (the *α* E chain of the *α*Eb7 integrin) small intestine lamina propria (siLP) DCs are potent inducers of homing receptors (CCR9 and the *α*4b7 integrin) on (CD4 and CD8) T and IgA+ B cells. These subsets have the enhanced capacity to induce the differentiation of Foxp3-expressing regulatory T (Treg) cells. In part, this process is driven by vitamin A metabolite retinoic acid [63], and it is crucial in the immunological tolerance to preserve tissue homeostasis.

CD4+ regulatory T cells are essential components of the host-microbiota symbiosis. The two main subtypes of T Reg cells are CD4+FoXP3+ T Reg cells that are found in the colon and small intestinal lamina propria and CD4+FoXP3–Il-10+ T Reg cells that are found in the small intestinal intraepithelial and lamina propria compartments [65]. Colonic FoXP3+ T Reg cells express Il-10, which reciprocally inhibits TH17 and TH1 cells.

### 2.3. Dysbiosis

The essential role of bacteria in the pathogenesis of colitis has achieved a general consensus. Although animal models of colitis do not mimic exactly Crohn's disease or ulcerative colitis, they provide one of the best evidences of the direct or indirect etiological role of bacteria in IBD. Human studies, however, have not been so conclusive. Even if these studies have failed to reveal categorically a specific altered composition on the microbial makeup of IBD patients versus control subjects, either in the stools or associated to biopsies, they support the hypothesis that a general “dysbiosis” underlies IBD. This term has been largely used since the late 50s and 60s to define deviations of the “normal” bacterial flora under antibiotic treatment in infants, adults, and patients in intensive care [66, 67]. In a healthy individual, the fecal bacterial community has a remarkable stability. This has been largely reported during the last years [68–71]. In contrast, a relatively unstable microbiota has been shown in Crohn's disease patients [72–74]. The presence of “unusual” bacteria was associated to the disease, for instance, low proportions of Firmicutes particularly *C. leptum*, high proportions of Gram-negative bacteria [75], conflicting information about *B. vulgatus* [74, 76], reduced concentration of *F. prausnitzii* [77], and increased numbers of Enterobacteria [72, 78]. Little information is available with regards to ulcerative colitis, but it seems to follow the same trend in that UC patients have a less diverse bacterial community [75, 79]. Pyrosequencing data of healthy and IBD patients show that the distribution of bacterial species differed only slightly between disease states (i.e., Crohn's disease versus ulcerative colitis) with regards to anatomic sites. The IBD group had marked decreases in the main representatives of the gut community: Bacteroidetes and Firmicutes [80].

Similarly, a dysbiosis was described on mucosal biopsies from IBD patients. Bacteria from these sites have been shown to differ from the luminal bacteria [81, 82].

The group of Schreiber [83] reported a reduction in the microbial diversity in mucosal specimens from Crohn's disease patients reflected in a reduction in the number of DGGE bands and in the diversity indices. Notwithstanding some contradictory results reported by different laboratories, a common finding seems to be an increased concentration of total bacteria both in CD and UC [30, 84–87].

Some reports argue against a localized dysbiosis to explain the patchy distribution of mucosal lesions [26, 88] or crypt abscesses [89]. One report suggests the contrary, but in this case biopsy samples from inflamed and noninflamed sites were pooled before the analysis [90].

In conclusion, despite the exponential evolution of technological approaches, the complex gut ecosystem still remains enigmatic. Efforts in deciphering its impact on IBD suggest that restoring shifts from the “normal” commensal microbiota rather than focusing on one particular member may improve these conditions.
